# Supplementary material for: Remodeling of maternal mRNA through poly(A) tail orchestrates human oocyte-to-embryo transition
Source: Nat Struct Mol Biol. 2023 Jan 16;30(2):200–15. doi: 10.1038/s41594-022-00908-2 (PMC9935398; doi:10.1038/s41594-022-00908-2)
Supplement: Supplementary file 1 — Reporting Summary [file 41594_2022_908_MOESM1_ESM.pdf]

nature portfolio

Corresponding author(s): Falong Lu
Last updated by author(s): Dec 2, 2022

Reporting Summary

Nature Portfolio wishes to improve the reproducibility of the work that we publish. This form provides structure for consistency and transparency in reporting. For further information on Nature Portfolio policies, see our Editorial Policies and the Editorial Policy Checklist.

Statistics

For all statistical analyses, confirm that the following items are present in the figure legend, table legend, main text, or Methods section.

n/a	Confirmed	
☐	☒	The exact sample size (*n*) for each experimental group/condition, given as a discrete number and unit of measurement
☒	☐	A statement on whether measurements were taken from distinct samples or whether the same sample was measured repeatedly
☐	☒	The statistical test(s) used AND whether they are one- or two-sided<br>*Only common tests should be described solely by name; describe more complex techniques in the Methods section.*
☒	☐	A description of all covariates tested
☐	☒	A description of any assumptions or corrections, such as tests of normality and adjustment for multiple comparisons
☐	☒	A full description of the statistical parameters including central tendency (e.g. means) or other basic estimates (e.g. regression coefficient) AND variation (e.g. standard deviation) or associated estimates of uncertainty (e.g. confidence intervals)
☐	☒	For null hypothesis testing, the test statistic (e.g. *F*, *t*, *r*) with confidence intervals, effect sizes, degrees of freedom and *P* value noted<br>*Give P values as exact values whenever suitable.*
☒	☐	For Bayesian analysis, information on the choice of priors and Markov chain Monte Carlo settings
☒	☐	For hierarchical and complex designs, identification of the appropriate level for tests and full reporting of outcomes
☐	☒	Estimates of effect sizes (e.g. Cohen's *d*, Pearson's *r*), indicating how they were calculated

Our web collection on statistics for biologists contains articles on many of the points above.

Software and code

Policy information about availability of computer code

Data collection	The sequencing data was acquired from Pacific BioSciences (PacBio) sequencing platform sequel II HiFi mode with the default parameters.
Data analysis	Ccs (version 5.0.0) was used to process subreads generated by PacBio SMRTbell sequencing.<br>The bam format reads were converted to fastq format using bam2fastq software in pbbam package (https://github.com/PacificBiosciences/pbbam, version 1.0.6).<br>The number of passes for each of the raw CCS read was generated using GetCCSpass.pl (https://github.com/Lulab-IGDB/polyA_analysis/blob/main/bin/).<br>samtools software (http://samtools.sourceforge.net, version 1.9).<br>Minimap2 (version v2.15) was used to align ccs reads to reference genome.<br>Read counts of each gene and gene assignments of each CCS reads were summarized by featureCounts v2.0.0.<br>Custom scripts used for data analysis are available in github: https://github.com/Lulab-IGDB/polyA_analysis.

For manuscripts utilizing custom algorithms or software that are central to the research but not yet described in published literature, software must be made available to editors and reviewers. We strongly encourage code deposition in a community repository (e.g. GitHub). See the Nature Portfolio guidelines for submitting code & software for further information.

Data

Policy information about availability of data

All manuscripts must include a data availability statement. This statement should provide the following information, where applicable:

- Accession codes, unique identifiers, or web links for publicly available datasets
- A description of any restrictions on data availability
- For clinical datasets or third party data, please ensure that the statement adheres to our policy

Data availability
The CCS data for human oocytes and embryos in bam format from PAIso-seq and PAIso-seq2 are available at Genome Sequence Archive for Human (GSA-Human: https://ngdc.cncb.ac.cn/gsa-human/) hosted by National Genomics Data Center (PAIso-seq: HRA001288, PAIso-seq2: HRA001289). Details for samples in HRA001288 and HRA001289 are shown in Supplementary Table 18. The bam files for visualization of the mapped reads in IGV are available at GSA-human (PAIso-seq: HRA001911, PAIso-seq2: HRA001912). The raw subreads data of FLAM-seq of Hela S3 cells, the human induced pluripotent stem cells (iPSCs), and human cerebral organoids were kindly provided by the original authors.
Genome and gene annotation
The genome sequence used in this study is from the following links: http://ftp.ebi.ac.uk/pub/databases/gencode/Gencode_human/release_36/GRCh38.primary_assembly.genome.fa.gz. The genome annotation used in this study is from the following links: http://ftp.ebi.ac.uk/pub/databases/gencode/Gencode_human/release_36/gencode.v36.primary_assembly.annotation.gtf.gz

Human research participants

Policy information about studies involving human research participants and Sex and Gender in Research.

Reporting on sex and gender	The oocytes used this study are from female donors, and the sperms used in this study are from male donors. The sex information for pre-implantation embryos has not been collected, as pre-implantation development is not likely affected by different sex.
Population characteristics	The donor women for oocytes are 25–38 years old with tubal-factor infertility and their partners have healthy semen. The donor men for sperms are men of normal semen of no more than 35 years old.
Recruitment	Immature oocytes in either the germinal vesicle (GV) or metaphase I (MI) stage were donated by patients taking intracytoplasmic sperm injection (ICSI) treatments, and these immature oocytes were not used in regular clinical practice. The donor women are 25–38 years old with tubal-factor infertility and their partners have healthy semen. In general, immature oocytes obtained in controlled ovarian hyperstimulation cycles were not used for subsequent clinical practice, because the development efficiency of embryos from immature oocytes was low, and patients generally had enough mature oocytes. Therefore, patients with a large number of follicles are communicated clearly about the research purpose in advance before oocyte retrieval to see if they are willing to donate immature oocytes for scientific research with no compensation, and also be ensured that the donated oocytes will be used for research only but not any clinical purposes. Written informed consent is signed by all the donors. When obviously immature GV or MI oocytes are identified by an embryologist during oocyte denuding, another embryologist will confirm the oocyte maturity and then check whether the patient has signed the informed consent for donation. The oocytes meeting the requirements will be collected for subsequent scientific research. The sperms are cryopreserved normal semen donated for research purpose from men of no more than 35 years old with written informed consent signed.
Ethics oversight	Institutional Review Board of Reproductive Medicine, Shandong University

Note that full information on the approval of the study protocol must also be provided in the manuscript.

Field-specific reporting

Please select the one below that is the best fit for your research. If you are not sure, read the appropriate sections before making your selection.

☒ Life sciences ☐ Behavioural & social sciences ☐ Ecological, evolutionary & environmental sciences

For a reference copy of the document with all sections, see nature.com/documents/nr-reporting-summary-flat.pdf

Life sciences study design

All studies must disclose on these points even when the disclosure is negative.

Sample size	No statistical methods were used to pre-determine sample sizes but our sample sizes are similar to those used in previous publications (DOI: 10.1038/nsmb.2660, 10.1038/nature12364).
Data exclusions	MI-7 in the PAIso-seq dataset was excluded in the analysis of individual MI replicates due to low number of reads. One of the 3′-dA replicate for PAIso-seq2 dataset was lost during library preparation and was not included in the analysis.

Replication	At least four biological replicates were performed for each PAIso-seq experiments. Two biological replicates were performed for each PAIso-seq2 experiments. However, one 3'-dA treated embryo PAIso-seq2 replicate was lost during library preparation. To minimize the use of human embryos and the one replicate of 3′-dA treated sample gives compelling results, we proceed with one replicate for 3′-dA treated human 1-cell embryos.
Randomization	The oocytes and embryos were randomly assigned to each experimental groups.
Blinding	Data collection and analysis were not performed blind to the conditions of the experiments. We are investigating transcriptome-wide molecular profiling of RNA poly(A) tails which could not be observed before the analysis of the collected data. Therefore, blinding is not necessary.

Reporting for specific materials, systems and methods

We require information from authors about some types of materials, experimental systems and methods used in many studies. Here, indicate whether each material, system or method listed is relevant to your study. If you are not sure if a list item applies to your research, read the appropriate section before selecting a response.

Materials & experimental systems

n/a	Involved in the study
☒	☐ Antibodies
☒	☐ Eukaryotic cell lines
☒	☐ Palaeontology and archaeology
☒	☐ Animals and other organisms
☒	☐ Clinical data
☒	☐ Dual use research of concern

Methods

n/a	Involved in the study
☒	☐ ChIP-seq
☒	☐ Flow cytometry
☒	☐ MRI-based neuroimaging
